# Ensemble epistasis: thermodynamic origins of nonadditivity between mutations

**DOI:** 10.1093/genetics/iyab105

**Published:** 2021-07-13

**Authors:** Anneliese J Morrison, Daria R Wonderlick, Michael J Harms

**Affiliations:** 1 Institute of Molecular Biology, University of Oregon, Eugene, OR 97403, USA; 2 Department of Chemistry and Biochemistry, University of Oregon, Eugene OR 97403, USA

**Keywords:** epistasis, thermodynamic ensemble, protein evolution, predictability, thermodynamics

## Abstract

Epistasis—when mutations combine nonadditively—is a profoundly important aspect of biology. It is often difficult to understand its mechanistic origins. Here, we show that epistasis can arise from the thermodynamic ensemble, or the set of interchanging conformations a protein adopts. *Ensemble epistasis* occurs because mutations can have different effects on different conformations of the same protein, leading to nonadditive effects on its average, observable properties. Using a simple analytical model, we found that ensemble epistasis arises when two conditions are met: (1) a protein populates at least three conformations and (2) mutations have differential effects on at least two conformations. To explore the relative magnitude of ensemble epistasis, we performed a virtual deep-mutational scan of the allosteric $Ca^{2+}$ signaling protein S100A4. We found that 47% of mutation pairs exhibited ensemble epistasis with a magnitude on the order of thermal fluctuations. We observed many forms of epistasis: magnitude, sign, and reciprocal sign epistasis. The same mutation pair could even exhibit different forms of epistasis under different environmental conditions. The ubiquity of thermodynamic ensembles in biology and the pervasiveness of ensemble epistasis in our dataset suggests that it may be a common mechanism of epistasis in proteins and other macromolecules.

## Teaser

Addressing the mechanistic origins of epistasis is critical to understanding how genotype determines phenotype. Here, Morrison, Wonderlick, and Harms investigate the plausibility of “ensemble epistasis.” Macromolecules often adopt a set of interchanging structures called a thermodynamic ensemble. Mutations can change the relative population of each structure, altering the effects of future mutations. The conditions that favor ensemble epistasis are common in macromolecules and the authors conclude that thermodynamic ensembles are a key source of intramolecular epistasis

## Introduction

Epistasis—when the effect of a mutation depends on the presence or absence of other mutations—is a common feature of biology. Epistasis can hint at biological mechanism (Maisnier-Patin *et al.* 2007; Ortlund *et al.* 2007; Alexander *et al.* 2009; Yokoyama *et al.* 2014; Baier *et al.* 2019; Yang *et al.* 2019), profoundly shape evolution (Weinreich *et al.* 2005; Poelwijk *et al.* 2007; Sailer and Harms 2017b), and complicate bioengineering that involves simultaneously introducing multiple mutations Giger *et al.* 2013; Sykora *et al.* 2014; Miton and Tokuriki 2016). It is therefore important to understand the general mechanisms by which epistasis can arise. Such knowledge will help us better understand biological systems, explain historical evolutionary trajectories, and improve models to predict the combined effects of mutations.

**Figure 1:**
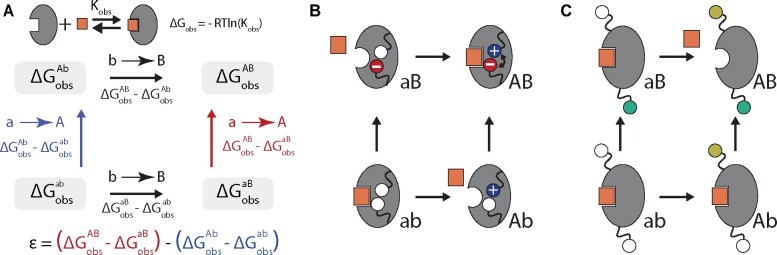
Mechanistic and mathematical descriptions of epistasis. (A) The mathematical description of epistasis (*ε*) in ligand binding free energy ($\Delta G_{\mathrm{obs}}$) for the mutant cycle between genotypes *ab* and *AB*. $\Delta G_{\mathrm{obs}}$ measures the strength of the binding interaction between protein (gray) and ligand (orange). We indicate genotypes as superscripts. *ε* is defined as the difference in the effect of mutation a→A in the *aB* background (red text), *vs* its effect in the *ab* background (blue text). (B) Mutant cycle where epistasis is readily understood: the a→A and b→B mutations introduce charges into the hydrophobic core, destabilizing the protein and disrupting binding of the orange square. Mutations a→A and b→B lead to a new electrostatic interaction when introduced together (minus and plus signs) restoring stability and binding. (C) Mutant cycle with difficult-to-understand epistasis. Mutations at two distant sites (green and yellow spheres) have no effect on binding of the orange square when introduced independently, but disrupt binding when introduced together.

One important class of epistasis is that which occurs between mutations within a single protein. The magnitude of such epistatic interactions, *ε*, can be quantitatively described as shown in Figure 1A; it simply represents the difference in the effect of mutation a→A in the *ab* and *aB* backgrounds. Sometimes, such epistasis can be understood intuitively. In Figure 1B, epistasis arises because the positive charge of mutation a→A is adjacent to the negative charge of mutation b→B. Epistasis occurs as a result of an electrostatic interaction between charged residues. Sometimes, however, epistasis can be difficult to rationalize. Figure 1C shows epistasis between two positions distant in the structure. Where does such epistasis come from? Can it be predicted from an understanding of protein biochemistry?

We and others noted previously that the thermodynamic ensemble of a protein could potentially give rise to nonadditive interactions between mutations (Ancel and Fontana 2000; Sailer and Harms 2017c). Proteins exist as ensembles of interchanging conformations, where the probability of seeing an individual conformation is determined by its relative energy. The functional output of a protein is averaged over the functional properties and populations of all individual ensemble conformations (Motlagh *et al.* 2014; Tsai and Nussinov 2014; Wei *et al.* 2016). Mutations can have different effects on each conformation, redistributing their relative probabilities in a nonlinear fashion. The effects of such mutations with respect to an observable would not sum additively, leading to *ensemble epistasis*.

Many important questions about ensemble epistasis remain unanswered. Under what conditions is ensemble epistasis expected to arise? Can it lead to different classes of evolutionarily relevant epistasis, that is, magnitude, sign, reciprocal-sign, and high-order? Is it plausible that such epistasis could occur in a real protein, rather than the highly simplified lattice models we used previously? And, finally, are there signals for ensemble epistasis that one might detect experimentally?

To address these questions, we set out to rigorously describe the thermodynamic and mechanistic basis for ensemble epistasis. We identified the minimal set of conditions that are necessary to observe ensemble epistasis: (1) a protein populates three or more conformations and (2) mutations have differential effects on two or more conformations within the ensemble. We found that this can lead to many types of epistasis, including magnitude, sign, reciprocal sign, and high-order epistasis. From structure-based calculations on the allosteric S100A4 protein, we predict that a large fraction of mutant pairs in real proteins will exhibit ensemble epistasis. We also found that varying the concentration of allosteric effectors could tune epistasis, suggesting one might experimentally detect ensemble epistasis by measuring epistasis at different concentrations of allosteric effectors. We conclude that ensemble epistasis is likely an important determinant of nonadditivity between mutations in proteins.

## Materials and methods

For the S100A4 epistasis analysis, we used three published structures for S100A4: the apo structure (PDB 1M31), the $Ca^{2+}$ bound structure (PDB 2Q91), and the structure bound to both $Ca^{2+}$ and a peptide extracted from Annexin A2 (PDB 5LPU). We removed all non-$Ca^{2+}$ small molecules (including waters) and edited the files to have an identical set of non-hydrogen atoms for the S100A4 chains (trimming any residues before alanine 2 and after phenylalanine 93 in the uniprot sequence, P26447). We arbitrarily selected the first NMR model for the apo structure. Using ROSETTA (Linux build 2018.33.60351), we generated five independent, pre-minimized structures for each of the conformations (*apo*, *ca*, and *capep*). We then used the “cartesian_ddg” binary to introduce each mutation three times into each of these five pre-minimized structures, yielding 15 calculated ΔG values for each mutation in each of the three conformations Park *et al.* (2016). Finally, we averaged the 15 values for each mutation in each conformation. We assumed the units of these ΔG values were in ${\text{kcal}\cdot \text{mol}}^{-1}$Alford *et al.* (2017).

For a given genotype, we described the free energy of the calcium-bound form as a function of calcium chemical potential ($\mu_{Ca^{2+}}$) with the expression $G_{ca}(\mu_{Ca^{2+}})=G_{ca}^{{}^{\circ}}-4\mu_{Ca^{2+}}$. $G_{ca}^{{}^{\circ}}$ is a constant describing both the relative stability of the “open” form of the protein relative to the “closed” form and the affinity of the open form for $Ca^{2+}$. We treated the free energy of the apo form as $G_{\mathrm{apo}}^{{}^{\circ}}(\mu_{Ca^{2+}})=G_{\mathrm{apo}}^{{}^{\circ}}$, where $G_{\mathrm{apo}}^{{}^{\circ}}$ measures the free energy of the apo form. For convenience, we set $G_{\mathrm{apo}}^{{}^{\circ},ab}=0\;{\text{kcal}\cdot \text{mol}}^{-1}$ and $G_{ca}^{{}^{\circ},ab}=10\;{\text{kcal}\cdot \text{mol}}^{-1}$ for $\mu =0\;{\text{kcal}\cdot \text{mol}}^{-1}$. This models the fact that, at some reference [$Ca^{2+}$], the “closed” form is favored over the “open” form. As [$Ca^{2+}$] increases, $G_{ca}(\mu_{Ca^{2+}})$ becomes more negative and eventually becomes more favorable than *G_apo_*. To verify that this result was not due to the choice of $G_{ca}^{{}^{\circ}}$, we re-ran our analysis for different values of $G_{ca}^{{}^{\circ}}$. We found that changing the value of $G_{ca}^{{}^{\circ}}$ has little impact on the magnitude of epistasis we observe. Its main effect is changing the $\mu_{Ca^{2+}}$ value at which the maximum magnitude of epistasis is observed (see Supplementary Figure S1).

We modeled the effects of mutations as changes to $G_{ca}^{{}^{\circ}}$ and $G_{\mathrm{apo}}^{{}^{\circ}}$. For the *Ab* genotype, for example, we would write:

(1) $$G_{ca}^{Ab}({µ}_{Ca^{2+}})=G_{ca}^{{}^{\circ}}-4\mu_{Ca^{2+}}+\delta G_{ca}^{a\to A}\;$$

(2) $$G_{\mathrm{apo}}^{Ab}=G_{\mathrm{apo}}^{{}^{\circ}}+\delta G_{\mathrm{apo}}^{a\to A}\;$$

(3) $$\begin{array}{c} \langle G_{ca,\mathrm{apo}}^{Ab}\rangle \left(\mu_{Ca^{2+}}\right)=-\mathrm{RTln}\left(e^{-(G_{ca}^{{}^{\circ}}-4\mu_{Ca^{2+}}+\delta G_{ca}^{a\to A})/RT}+e^{-(G_{\mathrm{apo}}^{{}^{\circ}}+\delta G_{\mathrm{apo}}^{a\to A})/RT}\right) \end{array}$$

where $\delta G_{ca}^{a\to A}$ and $\delta G_{\mathrm{apo}}^{a\to A}$ are the energetic effects of mutation a→A on the *ca* and *apo* conformations, respectively. See Supplementary Section S5 for further information, including a derivation of the model.

### Data availability

Supplementary files available at FigShare. The file “Supplementary derivations and proofs” has all referenced derivations and proofs in the text. Supplementary Figure S1 demonstrates that our epistatic analysis of human S100A4 is not sensitive to our assumptions about the affinity of the protein for calcium. Supplementary material is available at figshare: https://doi.org/10.25386/genetics.14377394. All analyses and ROSETTA input files can be downloaded directly from https://github.com/harmslab/ensemble_epistasis (last accessed July 19, 2021).

## Results

### Defining the three-conformation ensemble

**Figure 2:**
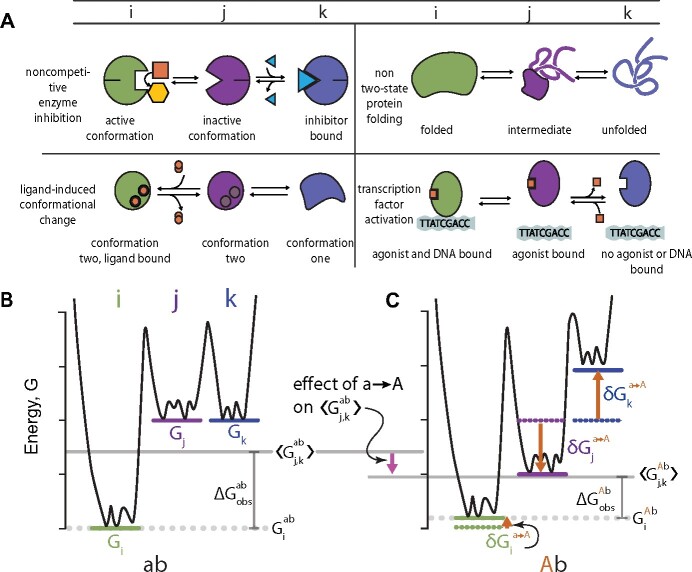
Mutations affect multiple ensemble conformations. (A) Schematic examples of biological mechanisms in which a protein populates at least three conformations. Columns indicate conformation labels—*i* (green), *j* (purple), or *k* (blue). (B) Energy diagram for a hypothetical protein with the *ab* genotype that adopts conformations *i* (green line), *j* (purple line), and *k* (blue line). The solid gray line indicates $\langle G_{j,k}^{ab}\rangle$ (the average energy of the inactive conformations *j* and *k*) and the dotted gray line indicates $G_{i}^{ab}$ (the energy of the active conformation *i*). The difference between the solid and dotted gray lines is the observable, $\Delta G_{\mathrm{obs}}^{ab}$. (C) Hypothetical mutation a→A changes the energies of conformations *i*, *j*, and *k* and thus $\Delta G_{\mathrm{obs}}$. Orange arrows represent the effect of mutation a→A on individual conformations. For example, $\delta G_{j}^{a\to A}$ shows the effect on conformation *j*. The mutation has a small effect on *i*, stabilizes *j*, and destabilizes *k*. This leads to a net decrease in $\langle G_{j,k}^{Ab}\rangle$ relative to $\langle G_{j,k}^{ab}\rangle$ (pink arrow), and thus a decrease in $\Delta G_{\mathrm{obs}}^{Ab}$ relative to $\Delta G_{\mathrm{obs}}^{ab}$.

To understand how the thermodynamic ensemble might lead to epistasis, we first defined a simple quantitative model of a protein exchanging between three conformations *i*, *j*, and *k*. We defined *i* as the “active” conformation in equilibrium with two “inactive” conformations *j* and *k*. This is a generic model that describes, in broad strokes, a wide variety of functions that depend on conformational change (Figure 2A). For example, conformation *i*, but not conformations *j* and *k*, could be capable of catalysis.

We will analyze epistasis in the free energy difference between the active *i* conformation and the inactive conformations, *j* and *k* ($\Delta G_{\mathrm{obs}}$). This quantifies how much the active form of the enzyme is favored over the inactive forms. We define $\Delta G_{\mathrm{obs}}$ as follows:

(4) $$\Delta G_{\mathrm{obs}}=G_{i}-\langle G_{j,k}\rangle ,$$

where *G_i_* is the energy of conformation *i* and $\langle G_{j,k}\rangle$ is the Boltzmann-weighted average of the free energies of conformations *j* and *k* (Figure 2B). Importantly, the free energy scale is linear, meaning—in the absence of epistasis—we expect the effects of mutations to sum.

We will now describe the origin of Equation (4) (some readers may wish to proceed to the next section *Mutations can affect multiple conformations in the ensemble*).

Due to thermal fluctuations, an individual protein molecule will flip between conformations *i*, *j*, and *k* over time. As a consequence, a population of many protein molecules will exhibit a mixture of conformations. Factors such as the number of favorable chemical bonds within each conformation determine the frequency of that conformation in the protein population.

The favorability of each conformation can be quantified by its free energy (*G*). Figure 2B shows a free energy landscape for a three-conformation ensemble. The large energy wells correspond to conformations *i*, *j*, and *k*, whereas the smaller wells correspond to small structural fluctuations within each conformation, such as side-chain rearrangements. Because conformation *i* has a low free energy in this hypothetical example, it will have a much higher frequency in the population than conformations *j* or *k*.

The statistical weight for a given conformation is related to its free energy by the Boltzmann distribution:

(5) $$w_{c}=e^{-G_{c}/RT}$$

where *c* indicates a conformation with free energy *G_c_*, *R* is the gas constant and *T* is the temperature in Kelvin. In the three-conformation ensemble, the frequency of conformation *i* is given by:

(6) $$f_{i}=\frac{w_{i}}{w_{i}+w_{j}+w_{k}}=\frac{e^{-G_{i}/RT}}{e^{-G_{i}/RT}+e^{-G_{j}/RT}+e^{-G_{k}/RT}}.$$

Importantly, the frequencies of the conformations are coupled. For example, making conformation *j* more stable (by decreasing *G_j_*) will lower *f_i_*, even if *G_i_* remains the same. This is because individual protein molecules will spend more time in conformation *j* and thus less time, on average, in conformation *i*.

As noted above, we are modeling an ensemble in which conformation *i* is active and conformations *j* and *k* are not. A typical way to quantify activity in such a system is with an equilibrium constant, describing the frequency of *i* relative to *j* and *k*:

(7) $$K_{\mathrm{obs}}=\frac{f_{i}}{f_{j}+f_{k}}=\frac{e^{-G_{i}/RT}}{e^{-G_{j}/RT}+e^{-G_{k}/RT}}.$$

Equilibrium constants follow a multiplicative scale, meaning that the effects of mutations are expected to multiply rather than add. We will take logarithm of *K_obs_* and place the observable on a free-energy scale, where—in the absence of epistasis—mutational effects are expected to add:

(8) $$\Delta G_{\mathrm{obs}}=-\mathrm{RTln}\left(K_{\mathrm{obs}}\right)=G_{i}+\mathrm{RTln}\left(e^{-G_{j}/RT}+e^{-G_{k}/RT}\right).$$

$\Delta G_{\mathrm{obs}}$ measures the difference in the free energy, at equilibrium, of the active *i* conformation and the inactive *j* and *k* conformations (Figure 2B). We will write the second term as:

(9) $$\langle G_{j,k}\rangle \equiv -\mathrm{RTln}\left(e^{-G_{j}/RT}+e^{-G_{k}/RT}\right)$$

where the brackets denote the Boltzmann-weighted average. This gives us, finally:

(10) $$\Delta G_{\mathrm{obs}}=G_{i}-\langle G_{j,k}\rangle .$$

### Mutations can affect multiple conformations in the ensemble

We next considered the effects of mutations. Because each conformation may have different physical interactions, the same mutation may have different effects on different conformations. For the three-conformation ensemble in Figure 2B, we thus need terms to describe the effect of the mutation on conformations *i*, *j* and *k*. To keep track of these effects, we will use the following notation:

- The observable energy for genotype *g* is $\Delta G_{\mathrm{obs}}^{g}$ (*e.g.*, $\Delta G_{\mathrm{obs}}^{ab}$).
- The energy of conformation *c* is $G_{c}^{g}$ (*e.g.*, $G_{i}^{ab}$).
- The energetic effect of mutation x→X on conformation *c* is $\delta G_{c}^{x\to X}$ (*e.g.*, $\delta G_{j}^{a\to A}$). Unless indicated, mutations are always introduced into the *ab* genetic background.
- Epistasis within a conformation—meaning the difference in the effect of a→A on the energy of conformation *c* in the *ab* and *aB* backgrounds—is $\delta \delta G_{c}^{ab\to AB}$.

We will now consider the effect of mutation a→A on $\Delta G_{\mathrm{obs}}^{ab}$ (Figure 2C). The three terms that describe its effect are $\delta G_{i}^{a\to A},\;\delta G_{j}^{a\to A}$, and $\delta G_{k}^{a\to A}$. Figure 2C shows how a hypothetical mutation a→A might change the ensemble: it has a small effect on conformation *i*, stabilizes *j*, and destabilizes *k*. We would describe the effect of the mutation mathematically as:

(11) $$\Delta G_{\mathrm{obs}}^{Ab}=\left(G_{i}^{ab}+\delta G_{i}^{a\to A}\right)-\langle G_{j,k}^{Ab}\rangle$$

where

(12) $$\begin{array}{c} \langle G_{j,k}^{Ab}\rangle \equiv -\mathrm{RTln}\left(e^{-(G_{j}^{ab}+\delta G_{j}^{a\to A})/RT}+e^{-(G_{k}^{ab}+\delta G_{k}^{a\to A})/RT}\right). \end{array}$$

The mutation in Figure 2C stabilizes $\langle G_{j,k}^{Ab}\rangle$ relative to $\langle G_{j,k}^{ab}\rangle$ because conformation *j* becomes so much more favorable. As a result, the $\Delta G_{\mathrm{obs}}^{Ab}$ is lower than $\Delta G_{\mathrm{obs}}^{ab}$ (Figure 2C).

The next step is to describe the effect of introducing two mutations simultaneously. To isolate epistasis that arises solely from changes to the thermodynamic ensemble, we will start by assuming that mutations are additive within each conformation. By this, we mean that $G_{c}^{AB}=G_{c}^{ab}+\delta G_{c}^{a\to A}+\delta G_{c}^{b\to B}$. There are no epistatic contributions of the form $\delta \delta G_{c}^{ab\to AB}$ reflecting physical interactions within each conformation of the sort seen in Figure 1B. This means any epistasis we observe arises solely from the ensemble. We will revisit this simplifying assumption later.

Using this framework, we can describe the combined effects of mutations a→A and b→B on $\Delta G_{\mathrm{obs}}$ as the following:

(13) $$\Delta G_{\mathrm{obs}}^{AB}=\left(G_{i}^{ab}+\delta G_{i}^{a\to A}+\delta G_{i}^{b\to B}\right)-\langle G_{j,k}^{AB}\rangle$$

where

(14) $$\begin{array}{c} \langle G_{j,k}^{AB}\rangle \equiv -\mathrm{RTln}\left(e^{-(G_{j}^{ab}+\delta G_{j}^{a\to A}+\delta G_{j}^{b\to B})/RT}+e^{-(G_{k}^{ab}+\delta G_{k}^{a\to A}+\delta G_{k}^{b\to B})/RT}\right) \end{array}.$$

### The thermodynamic ensemble can lead to epistasis

To understand the nature of epistasis arising from such a system, we must map the thermodynamic model in Equation (13) to epistasis. Table 1 shows the mapping between each genotype and its thermodynamic description, $\Delta G_{\mathrm{obs}}^{\mathrm{genotype}}$. We will treat epistasis as the quantitative difference between the effects of mutation a→A in the *ab* and *aB* backgrounds (Figure 1A):

(15) $$\varepsilon =\left(\Delta G_{\mathrm{obs}}^{AB}-\Delta G_{\mathrm{obs}}^{aB}\right)-\left(\Delta G_{\mathrm{obs}}^{Ab}-\Delta G_{\mathrm{obs}}^{ab}\right).$$

**Table 1:** ** Map between genotype and the thermodynamic description of** $\Delta G_{\mathrm{obs}}^{\mathrm{genotype}}$

Genotype	$\Delta G_{\mathrm{obs}}^{\mathrm{genotype}}$	$\langle G_{j,k}^{\mathrm{genotype}}\rangle$
*ab*	$G_{i}^{ab}-\langle G_{j,k}^{ab}\rangle$	$-\mathrm{RTln}\left(e^{-\left(G_{j}^{ab}\right)/RT}+e^{-\left(G_{k}^{ab}\right)/RT}\right)$
*Ab*	$(G_{i}^{ab}+\delta G_{i}^{a\to A})-\langle G_{j,k}^{Ab}\rangle$	$-\mathrm{RTln}\left(e^{-\left(G_{j}^{ab}+\delta G_{j}^{a\to A}\right)/RT}+e^{-\left(G_{k}^{ab}+\delta G_{k}^{a\to A}\right)/RT}\right)$
*aB*	$(G_{i}^{ab}+\delta G_{i}^{b\to B})-\langle G_{j,k}^{aB}\rangle$	$-\mathrm{RTln}\left(e^{-\left(G_{j}^{ab}+\delta G_{j}^{b\to B}\right)/RT}+e^{-\left(G_{k}^{ab}+\delta G_{k}^{b\to B}\right)/RT}\right)$
*AB*	$(G_{i}^{ab}+\delta G_{i}^{a\to A}+\delta G_{i}^{b\to B})-\langle G_{j,k}^{AB}\rangle$	$-\mathrm{RTln}\left(e^{-\left(G_{j}^{ab}+\delta G_{j}^{a\to A}+\delta G_{j}^{b\to B}\right)/RT}+e^{-\left(G_{k}^{ab}+\delta G_{k}^{a\to A}+\delta G_{k}^{b\to B}\right)/RT}\right)$

We can substitute the thermodynamic equations for each $\Delta G_{\mathrm{obs}}$ from Table 1 into Equation (15). Upon simplifying this expression (Supplementary Section S1.1), we obtain:

(16) $$\varepsilon =-\left[\left(\langle G_{j,k}^{AB}\rangle -\langle G_{j,k}^{aB}\rangle \right)-\left(\langle G_{j,k}^{Ab}\rangle -\langle G_{j,k}^{ab}\rangle \right)\right].$$

All terms associated with conformation *i* cancel. We are left with a description of *ε* that is only in terms of mutational effects on conformations *j* and *k*.

Our expression for *ε* is determined by the effects of mutations a→A and b→B on conformations *j* and *k*, not their effects on conformation *i*. Perturbations to the relative populations of *j* and *k* necessarily lead to nonlinear changes in $\Delta G_{\mathrm{obs}}$ because the logarithmic term in $\langle G_{j,k}\rangle$ cannot be simplified further.

### Conditions necessary for ensemble epistasis

We next used the thermodynamic description of ensemble epistasis derived above (Equation 16) to ask under what conditions ensemble epistasis is expected to arise. In the Supplementary Text, we show that there are two necessary conditions for ensemble epistasis:

1. The protein populates at least three conformations (Supplementary Section S1.2)
2. Mutations have differential effects on conformations *j* and *k* (Supplementary Section S1.3).

**Figure 3:**
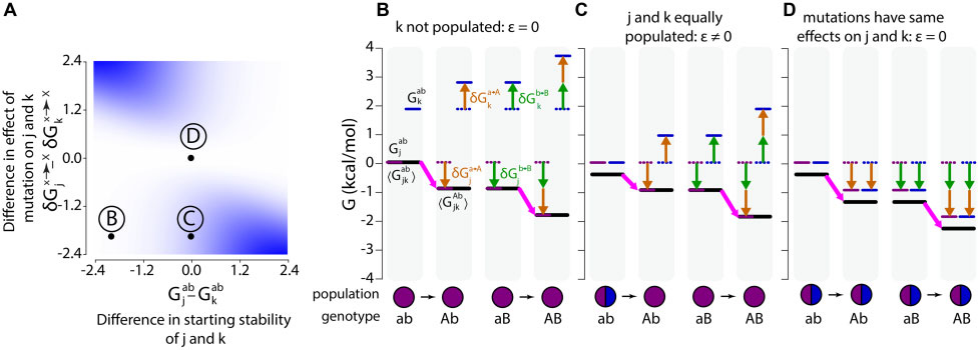
Ensemble epistasis arises from redistributed conformational probabilities. (A) Epistasis as a function of the difference in the effects of the mutations a→A and b→B on conformations *j* and *k* ($\delta G_{j}^{x\to X}-\delta G_{k}^{x\to X}$ in ${\text{kcal}\cdot \text{mol}}^{-1}$, y-axis) and the difference in the stability of conformations *j* and *k* for the *ab* genotype ($G_{j}^{ab}-G_{k}^{ab}$ in ${\text{kcal}\cdot \text{mol}}^{-1}$, x-axis). Color indicates the magnitude of epistasis, ranging from 0 (white) to $1.6\;{\text{kcal}\cdot \text{mol}}^{-1}$ (blue). For the whole plot, a→A and b→B had identical effects ($\delta G_{j}^{a\to A}=\delta G_{j}^{b\to B}$ and $\delta G_{k}^{a\to A}=\delta G_{k}^{b\to B}$). We set $G_{j}^{ab}=0\;{\text{kcal}\cdot \text{mol}}^{-1}$ and $\delta G_{j}^{x\to X}=-0.96\;{\text{kcal}\cdot \text{mol}}^{-1}$ and then varied $G_{k}^{ab}$ and $\delta G_{k}^{x\to X}$ to sample parameter space. All calculations were done at T = 298 K. (B–D) The thermodynamic origins for the epistasis at points *B*, *C*, and *D* are indicated on (A). The color scheme is consistent throughout: purple and blue lines are the energies of conformations *j* and *k*, respectively; orange arrows show the effects of mutation a→A; green arrows show the effects of mutation b→B; heavy black lines are the Boltzmann-weighted average energies of *j* and *k*, $\langle G_{j,k}\rangle$; heavy pink arrows are the observed effect of mutation a→A in the genotype indicated below the plot. The difference between the length of the pink arrows in the ab→Ab and aB→AB genotypes measures *ε*.The relative populations of conformations *j* and *k* are shown as a pie chart below the energy diagram.

To understand what these conditions mean in practice, we calculated ensemble epistasis using Equation (16) as a function of the difference in the stabilities of conformations *j* and *k* ($G_{j}^{ab}-G_{k}^{ab}$) and the difference in the effects of mutations on conformations *j* and *k* ($\delta G_{j}^{x\to X}-\delta G_{k}^{x\to X}$) (Figure 3A). In Figure 3, B–D, we reveal the underlying ensemble that leads to the epistasis observed in Figure 3A. The length of the pink arrows illustrates the effect of mutation a→A in each genetic background, *ab* or *aB*. The difference in the length of the pink arrows for the ab→Ab and aB→AB genotypes measures epistasis, *ε*.

We can see why multiple conformations are required for ensemble epistasis by comparing points *B* and *C* on Figure 3A. At point *B*, only conformation *j* is appreciably populated for all genotypes (pie charts, Figure 3B); at point *C*, conformations *j* and *k* have equal starting populations (pie charts, Figure 3C). This difference in the starting populations of *j* and *k* leads to different epistatic outcomes. At point *B*, both ab→Ab and aB→AB depend only on the effect of the mutation on conformation *j* because it is the only conformation appreciably populated. The lengths of the pink arrows are equal, indicating that there is no epistasis. At point *C*, the effect of ab→Ab on $\langle G_{j,k}\rangle$ is moderate because the stabilization of conformation *j* is offset by the entropic cost of depopulating conformation *k*. This results in epistasis because when a→A is introduced into the *aB* background, mutation b→B has already depopulated conformation *k*. As a result, the effect of aB→AB is determined solely by its stabilization of conformation *j*, and is thus larger than ab→Ab.

We can see why differential effects for each mutation are required by comparing points *C* and *D* on Figure 3A. At both points, conformations *j* and *k* have equal starting populations (pie charts, Figure 3, C and D). At point *C*, the mutations have opposite effects on conformations *j* and *k* (Figure 3C); at point *D*, the mutations have identical effects on conformations *j* and *k* (Figure 3D). This means that for point *D* the introduction of a→A or b→B shifts the total energy landscape, but does not change the relative proportions of *j* and *k*. As a result, mutation a→A has the same effect regardless of background (compare pink arrows, Figure 3D).

### Ensembles can lead to magnitude epistasis, sign-epistasis, and reciprocal sign-epistasis

We next asked if the ensemble could lead to different evolutionarily relevant classes of epistasis: magnitude, sign, and reciprocal sign epistasis. In magnitude epistasis, only the magnitude of a mutation’s effect changes when another mutation is introduced. In sign epistasis, the same mutation has a positive effect in one background and a negative effect in another. Finally, in reciprocal sign epistasis, both mutations exhibit sign epistasis.

**Figure 4:**
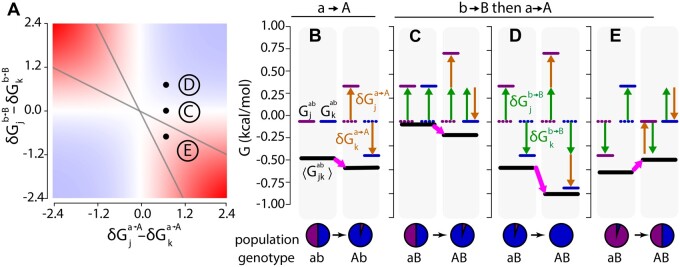
Ensemble epistasis arises when mutations have different effects on different conformations. (A) Epistasis calculated for a three-conformation ensemble that starts with $G_{j}^{ab}=G_{k}^{ab}=0$. The differences in the effects of mutations a→A and b→B on conformations *j* and *k* are indicated on the x- and y-axes. The magnitude of epistasis is indicated by the color, ranging from + 1.6 (dark red) to 0 (white) to $-1.6\;\mathrm{kcal}\cdot mol^{-1}$ (dark blue). Gray lines delineate regions of reciprocal sign (red regions within the lines) and sign epistasis (red regions outside of the lines). All calculations were done at T=298 K. (B–E) The thermodynamic origins of the epistasis indicated by points *C*, *D*, and *E* on (A). The effect of mutation a→A is constant in all panels; the effect of mutation b→B differs depending on the scenario. The color scheme is consistent with Figure 2. (B) The effect of a→A in the *ab* background. a→A destabilizes *j* and stabilizes *k*, stabilizing $\langle G_{j,k}^{AB}\rangle$. (C) Scenario C: no epistasis. b→B has the same effect on conformations *j* and *k*. (D) Scenario D: a→A and b→B act synergistically to destabilize *j* and stabilize *k*. (E) Scenario E: a→A and b→B have opposite effects on conformations *j* and *k*.

We surveyed the parameter space for the effects of mutations on each conformation while tracking the magnitude and type of epistasis observed (Figure 4A). We set the initial energies of conformations *j* and *k* to be equal ($G_{j}^{ab}=G_{k}^{ab}=0$). We then calculated epistasis using Equation (16) as a function of the difference in the effects of mutations a→A and b→B on *j* and *k*.

We found four regimes, corresponding to magnitude, sign, reciprocal sign, and no epistasis. To understand the origins of these three regimes, we studied the thermodynamic ensembles that lead to epistasis at the points indicated *C*, *D*, and *E*. At this slice of parameter space, mutation a→A destabilizes conformation *j* by $0.35\;\mathrm{kcal}\cdot mol^{-1}$ and stabilizes conformation *k* by $-0.35\;\mathrm{kcal}\cdot mol^{-1}$. The effect of this mutation on the ensemble in the *ab* background is shown in Figure 4B: the mutation mildly stabilizes $\langle G_{j,k}\rangle$.

At point *C*, we see no epistasis (Figure 4A). We can see why this occurs in Figure 4C. Mutation b→B destabilizes both *j* and *k* by $0.35\;\mathrm{kcal}\cdot mol^{-1}$. Because mutation b→B does not have differential effects on each conformation, $\langle G_{j,k}\rangle$ is globally shifted by $+0.35\;\mathrm{kcal}\cdot mol^{-1}$. Introducing a→A and b→B together yields no epistasis because both the *ab* and *aB* genotypes have identical configurations—the observed effect comes only from mutation a→A (compare pink arrows in Figure 4, B and C).

At point *D*, we observe magnitude epistasis (Figure 4A). We can see why this occurs in Figure 4D. Mutations a→A and b→B have synergistic effects on each conformation: *k* is stabilized while *j* is destabilized. We see magnitude epistasis because although the relative population of *j* is reduced, it still has weight in the Boltzmann-weighted average stability (compare pink arrows in Figure 4, B and D).

At point *E*, we see reciprocal sign epistasis (Figure 4A). We can see why this occurs in Figure 4E. a→A and b→B have opposite effects on *j* and *k*: a→A destabilizes *j* and stabilizes *k*, whereas b→B stabilizes *j* and destabilizes *k*. The effects are equal in magnitude but opposite in sign so their combined effects cancel, yielding $\langle G_{j,k}^{AB}\rangle$ equal to that of the *ab* genotype (compare pink arrows in Figure 4, B and E). As a result, mutations a→A and b→B have individually stabilizing effects on $\langle G_{j,k}\rangle$ but are destabilizing when combined.

The magnitude and sign regions of Figure 4A show distinct patterns with regard to the sign of epistasis observed: mutations in the magnitude region are more stabilizing (positive epistasis) and those in the sign region are more destabilizing (negative epistasis) than anticipated based on single mutational effects. The magnitude region results in positive epistasis because mutations work synergistically to hyper-stabilize one conformation, while greatly destabilizing the other. This results in one conformation having very little weight in the Boltzmann distribution such that the remaining stabilized conformation determines the observable value. In the sign region, each mutation preferentially stabilizes a different conformation when introduced alone. However, when introduced together, they have opposing effects within a single conformation. The stabilizing effects of each mutation alone on $\langle G_{j,k}\rangle$ cancel, resulting in a less stable double mutant than anticipated.

### The thermodynamic ensemble can lead to high-order epistasis

In addition to magnitude, sign, and reciprocal sign epistasis, high-order epistasis is evolutionarily important (Weinreich *et al.* 2013; Sailer and Harms 2017b). In high-order epistasis, the effect of a three-way mutant cannot be explained by the individual and pairwise effects of its constituent mutations. In the supplement we find that high-order epistasis may arise by redistributing the relative populations of conformations *j* and *k* (see Supplementary Section S2). We anticipate that the results we have found for pairwise epistasis—the importance of differential mutational effects on different conformations, for example—will apply to high-order ensemble epistasis, but further work is needed to clarify the necessary and sufficient conditions to observe high-order ensemble epistasis.

### Ensemble epistasis is not due to simplifying assumptions

We next wanted to relax two major assumptions we made above. The first assumption was that there were no epistatic interactions within conformations (as in Figure 1B). We show in the Supplementary Section S3 that epistasis within each conformation can coexist alongside ensemble epistasis. We also revisit this question empirically in the following section, finding that ensemble epistasis and within-conformation epistasis have similar magnitudes.

The second assumption made above was that the ensemble could be described with only three conformations *i*, *j* and *k* (Figure 2). We asked what the form of ensemble epistasis would be if we considered an equilibrium between two sub-ensembles, *X* and *Y*, each of which could have many different conformations. The free energy difference between these sub-ensembles would be given by:

(17) $$\Delta G_{\mathrm{obs}}=\left[-\mathrm{RTln}\left(\sum_{m\in X}e^{-G_{m}/RT}\right)\right]-\left[-\mathrm{RTln}\left(\sum_{n\in Y}e^{-G_{n}/RT}\right)\right]$$

where *m* indexes over all conformations in *X* and *n* indexes over all conformations in *Y*. In more compact form, this would be:

(18) $$\Delta G_{\mathrm{obs}}=\langle G_{X}\rangle -\langle G_{Y}\rangle .$$

We show in the Supplementary Section S4 that for such a system, epistasis becomes:

(19) $$\begin{array}{c} \varepsilon =\left[\left(\langle G_{X}^{AB}\rangle -\langle G_{X}^{aB}\rangle \right)-\left(\langle G_{X}^{Ab}\rangle -\langle G_{X}^{ab}\rangle \right)\right]-\\ \left[\left(\langle G_{Y}^{AB}\rangle -\langle G_{Y}^{aB}\rangle \right)-\left(\langle G_{Y}^{Ab}\rangle -\langle G_{Y}^{ab}\rangle \right)\right]. \end{array}$$

Thus, we expect to see ensemble epistasis in such a system—for certain conformational energies and mutational effects, at least—because we cannot simplify the expression for *ε* further.

### Ensemble epistasis may be a common feature in protein mutant cycles

Above we showed mathematically that ensemble epistasis can arise when multiple conformations are populated and mutations have different effects on different conformations. We next wanted to address whether these requirements are met in real systems. Multi-conformation ensembles are common in biology and we expect that the first requirement is often met (Figure 2A). However, it is not obvious that the requirement for differential effects of mutations is commonly satisfied. We designed a computational test to ask if it was plausible that both of these conditions are met simultaneously in a protein.

**Figure 5:**
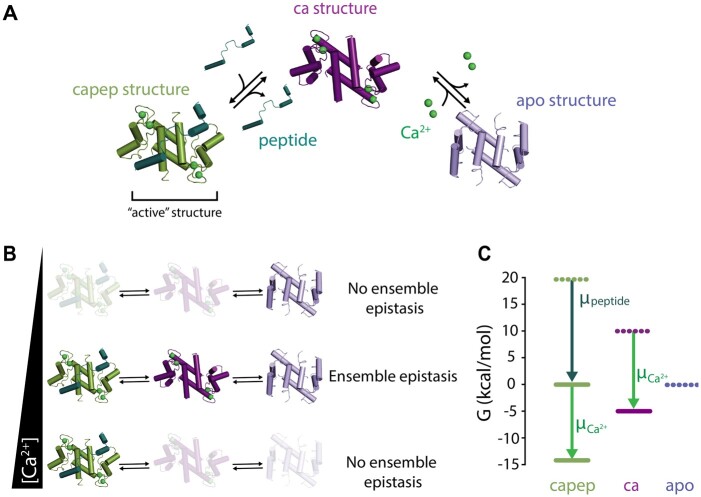
Testing for ensemble epistasis in the S100A4 protein. (A) Three-conformation ensemble of the S100A4 protein. The apo conformation (*apo*, slate, PDB: 1M31) is in equilibrium with the $Ca^{2+}$ bound (*ca*, purple, PDB: 2Q91) and $Ca^{2+}$/*peptide* bound (*capep*, green, PDB: 5LPU) conformations when $Ca^{2+}$ (lime green spheres) and *peptide* (dark green) are present. (B) The relative populations of the *apo*, *ca*, and *capep* conformations change as $Ca^{2+}$ concentration increases in the presence of saturating peptide. The magnitude of ensemble epistasis observed is $Ca^{2+}$-dependent, because only some $Ca^{2+}$ concentrations lead to multiple populated conformations. (C) Assigned energies ($\mathrm{kcal}\cdot mol^{-1}$) of S100A4 conformations. *Apo* is most stable when *peptide*, *μ_peptide_*, and $Ca^{2+}$ chemical potentials, $\mu_{Ca^{2+}}$, are zero (dashed lines). *Capep* is stabilized by increasing $\mu_{\mathrm{peptide}}=20\;{\text{kcal}\cdot \text{mol}}^{-1}$ (dark green arrow, solid green line). Increasing $\mu_{Ca^{2+}}$ alters the energies of both *ca* and *capep* (lime green arrow, solid lines). All calculations were done at T=298 K.

We investigated these questions using the allosteric $Ca^{2+}$ signaling protein, human S100A4. S100A4 adopts a three-conformation ensemble, meeting our first requirement to observe ensemble epistasis (Figure 5A; Vallely *et al.* 2002; Malashkevich *et al.* 2008; Ecsédi *et al.* 2017). In the absence of $Ca^{2+}$, it favors the “*apo*” conformation (Figure 5A, slate); addition of $Ca^{2+}$ stabilizes the “*ca*” conformation with an exposed hydrophobic peptide-binding surface (Figure 5A, purple); finally, addition of *peptide* leads to formation the “*capep*” conformation that has both $Ca^{2+}$ and *peptide* bound (Figure 5A, green). These structures can be assigned indices, as in our analytical model: *capep* (*i*), *ca* (*j*), and *apo* (*k*).

We used software for structure-based energy calculations (ROSETTA) to estimate the stability effects of all 3382 possible single point mutations to the *capep*, *ca*, and *apo* conformations of S100A4. This gives us $\delta G_{\mathrm{capep}}^{x\to X},\;\delta G_{ca}^{x\to X}$, and $\delta G_{\mathrm{apo}}^{x\to X}$ for every mutation x→X.

We then exploited the allosteric nature of S100A4 to switch between conditions where only single conformations are appreciably populated and where multiple conformations are populated. To model the ensemble, we selected reference concentrations of $Ca^{2+}$ and peptide such that $G_{\mathrm{capep}}^{{}^{\circ}}$≫$G_{ca}^{{}^{\circ}}$≫$G_{\mathrm{apo}}^{{}^{\circ}}$ (Figure 5C; see *Materials and Methods*). We know experimentally that the protein favors the *apo* conformation in the absence of $Ca^{2+}$ and *peptide* (Garrett *et al.* 2008). We modeled the signaling behavior of S100A4 by changing the concentrations of $Ca^{2+}$ and *peptide*: $G_{\mathrm{capep}}=G_{\mathrm{capep}}^{{}^{\circ}}-4\mu_{Ca^{2+}}-\mu_{\mathrm{peptide}}$ and $G_{ca}=G_{ca}^{{}^{\circ}}-4\mu_{Ca^{2+}}$, where $\mu_{Ca^{2+}}$ and μ*_peptide_* are the chemical potentials of $Ca^{2+}$ and *peptide* relative to their reference concentrations (Figure 5C). Depending on our choice of μCa2+ and μpeptide, we can observe different relative populations of the *capep*, *ca*, and *apo* conformations. For $\Delta G_{\mathrm{obs}}$, we used:

(20) $$\Delta G_{\mathrm{obs}}^{\mathrm{genotype}}=G_{\mathrm{capep}}^{\mathrm{genotype}}+\mathrm{RTln}\left(e^{-G_{ca}^{\mathrm{genotype}}/RT}+e^{-G_{\mathrm{apo}}^{\mathrm{genotype}}/RT}\right).$$

By analogy to what we derived in Equation (16), epistasis is calculated as:

(21) $$\varepsilon =-\left[\left(\langle G_{ca,\mathrm{apo}}^{AB}\rangle -\langle G_{ca,\mathrm{apo}}^{aB}\rangle \right)-\left(\langle G_{ca,\mathrm{apo}}^{Ab}\rangle -\langle G_{ca,\mathrm{apo}}^{ab}\rangle \right)\right].$$

We constructed all 5.6 million pairs of mutations by treating the $\delta G_{\mathrm{capep}}^{x\to X},\;\delta G_{ca}^{x\to X}$, and $\delta G_{\mathrm{apo}}^{x\to X}$ ROSETTA values as additive within each conformation, meaning that we calculated the effect of two mutations a→A and b→B in combination on the *apo* conformation, for example, as $G_{\mathrm{apo}}^{AB}=G_{\mathrm{apo}}^{ab}+\delta G_{\mathrm{apo}}^{a\to A}+\delta G_{\mathrm{apo}}^{b\to B}$. We made this assumption to isolate epistasis arising solely from changes to the ensemble, as we did in our general thermodynamic model in Equation (13).

**Figure 6:**
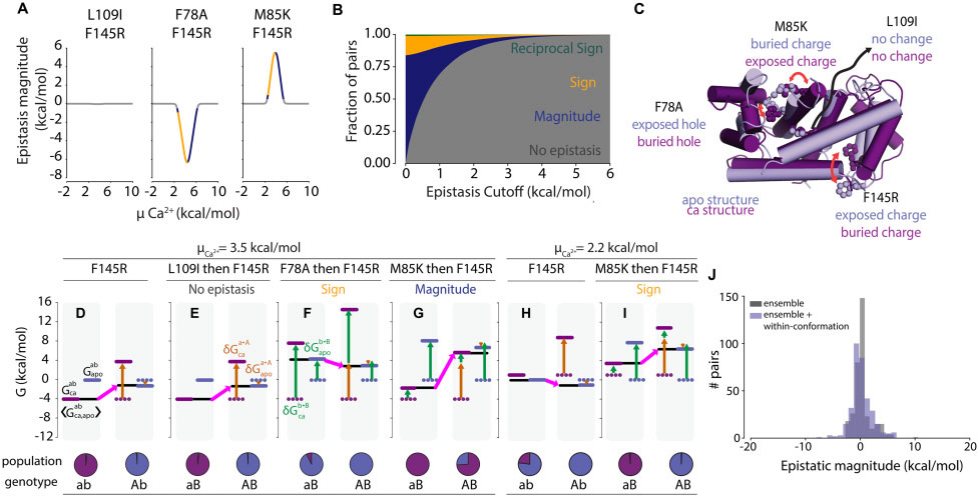
The ensemble of S100A4 exhibits ensemble epistasis. (A) Epistatic magnitude (${\text{kcal}\cdot \text{mol}}^{-1}$, y-axis) as a function of $\mu_{Ca^{2+}}({\text{kcal}\cdot \text{mol}}^{-1}$, x-axis) for three mutation pairs: L109I/F145R (left panel), F78A/F145R (middle panel), and M85K/F145R (right panel). Color is consistent with epistatic type in (B). (B) Fractional contribution of each epistatic type (y-axis) as a function of epistatic magnitude cutoff (${\text{kcal}\cdot \text{mol}}^{-1}$, x-axis), colored by type: reciprocal sign (green), sign (gold), and magnitude (dark blue). Pairs with epistasis below the cutoff are considered non-epistatic (gray). (C) Positions of mutations in the *ca* (purple) and *apo* (slate) conformations. Text indicates their relative environments in each conformation. Red arrows indicate changes in position between the *ca* and *apo* conformations. (D–I) Thermodynamic origins of epistasis for three mutation pairs at $\mu_{Ca^{2+}}=3.5\;{\text{kcal}\cdot \text{mol}}^{-1}$ (D–G) or $\mu_{Ca^{2+}}=2.2\;{\text{kcal}\cdot \text{mol}}^{-1}$ (H and I). $Ca^{2+}$ chemical potential is indicated above the panel. Mutation a→A (F145R) is constant; mutation b→B differs in (E–G and I). The color scheme is consistent throughout: purple and blue lines are the energies of *ca* and *apo*, respectively, whereas black lines represent $\langle G_{ca,\mathrm{apo}}^{\mathrm{genotype}}\rangle$; all other colors are consistent with Figures 2 and 3. Specific mutations and epistatic classes are indicated at the top of the panel; genotypes and relative populations are below. (G) Introduction of mutation F145R (a→A) into the *ab* background at $\mu_{Ca^{2+}}=3.5\;{\text{kcal}\cdot \text{mol}}^{-1}$. (E) No epistasis scenario: mutations F145R (a→A) and L109I (b→B). (F) Sign epistasis scenario: mutations F145R (a→A) and F78A (b→B). (G) Magnitude epistasis scenario: mutations F145R (a→A) and M85K (b→B). (H) Introduction of mutation F145R (a→A) into the *ab* background at $\mu_{Ca^{2+}}=2.2\;{\text{kcal}\cdot \text{mol}}^{-1}$ (I) Sign epistasis scenario: mutations F145R (a→A) and M85K (b→B). J) Histogram showing the distribution of epistasis between 344 mutant pairs assuming no epistasis between mutations within each conformation (gray), or using calculated epistasis between mutations within each conformation (slate blue).

Under the assumption of within-conformation additivity, we calculated epistasis in $\langle G_{ca,\mathrm{apo}}\rangle$ using Equation (21) as a function of $\mu_{Ca^{2+}}$ at a fixed *μ_peptide_* (see methods for more details). We observed peaks in epistasis at intermediate values of $\mu_{Ca^{2+}}$, where the *capep*, *ca*, and *apo* conformations may all be populated. In contrast, we observed no epistasis at low $\mu_{Ca^{2+}}$ (where only the *apo* conformation is populated) or high $\mu_{Ca^{2+}}$ (where only the *capep* conformation is populated). We observed three basic patterns of $\mu_{Ca^{2+}}$-dependent epistatic magnitude, as exemplified by the three mutant pairs shown in Figure 6A: F145R/L109I had no epistasis (left panel) while F145R/F78A had negative epistasis (middle panel) and F145R/M85K had positive epistasis (right panel). Interestingly, the type of epistasis observed—magnitude (dark blue), sign (gold), or reciprocal sign (green)—was also dependent upon $\mu_{Ca^{2+}}$ (Figure 6A). This was quite common in our dataset: ∼61% of pairs with an epistatic magnitude above $0.6\;{\text{kcal}\cdot \text{mol}}^{-1}$ switched epistatic type at least once as $\mu_{Ca^{2+}}$ increased.

We next looked at the magnitude and type of epistasis for all 5.6 million mutation pairs at their peak values over the range of $\mu_{Ca^{2+}}$. We found that 47% of the 5.6 million pairs exhibited epistasis at or above the order of thermal fluctuation, $0.6\;{\text{kcal}\cdot \text{mol}}^{-1}$ (Figure 6B). We found that 34% of pairs exhibited magnitude, 12% sign, and 1% reciprocal-sign epistasis at this cutoff. Approximately 11% of pairs exhibited epistasis with a magnitude above $2\;{\text{kcal}\cdot \text{mol}}^{-1}$.

To understand the structural origins of the observed epistasis, we compared the positions of each mutation from Figure 6A in the *apo* (slate, Figure 6C) and *ca* (purple, Figure 6C) conformations. We first consider F145R. This position is solvent exposed in the *apo* conformation but buried in the *ca* conformation. As a consequence, introducing Arg mildly stabilizes the *apo* conformation, but dramatically destabilizes the *ca* conformation due to burying its charge. Next, L109I is a conservative mutation at a site whose environment is essentially unchanged between the *apo* and *ca* conformations. F78A is solvent exposed in the *apo* conformation but buried in the *ca* conformation. The Phe to Ala mutation is destabilizing to the *ca* conformation due to the loss of hydrophobic contacts. Finally, M85K is buried in the *apo* conformation, but exposed in the *ca* conformation. Mutation to Lys introduces a buried charge, greatly destabilizing it due to the cost of ion desolvation. The differences in the effects of L109I, F78A, and M85K on the *apo* and *ca* conformations cause them to exhibit different types of epistasis when paired with F145R.

F145R exhibits no epistasis when paired with L109I at $\mu_{Ca^{2+}}=3.5\;{\text{kcal}\cdot \text{mol}}^{-1}$ (Figure 6E). The L109I mutation has a negligible effect on the *apo* and *ca* conformations (genotype *aB*, Figure 6E). As a result, F145R has the same effect on $\langle G_{ca,\mathrm{apo}}\rangle$ when introduced into both *ab* and L109I (*aB*) backgrounds (compare pink arrows in Figure 6, D and E).

Pairing F145R with F78A results in sign epistasis. F78A is destabilizing to both conformations, but much more so to the *ca* conformation (genotype *aB*, Figure 6F). Both F78A and F145R preferentially destabilize the *ca* structure, leading to a dramatic decrease in its relative population when introduced together (green arrows, Figure 6F). We see sign epistasis because the synergistic destabilization of the *ca* conformation makes $\langle G_{ca,\mathrm{apo}}^{AB}\rangle$ only dependent on the stability of the *apo* conformation (compare pink arrows in Figure 6, D and F).

F145R exhibits magnitude epistasis when paired with M85K. The M85K mutation is greatly destabilizing to the *apo* conformation and slightly destabilizing to the *ca* conformation (green arrows, Figure 4G). Combining both mutations causes a decrease in the stability of both conformations and a net destabilization of $\langle G_{ca,\mathrm{apo}}^{AB}\rangle$, leading to the observation of magnitude epistasis (pink arrows, Figure 6G).

Intriguingly, a slight decrease from $\mu_{Ca^{2+}}=3.5\;\text{to}\;2.2\;\mathrm{kcal}\cdot mol^{-1}$ switches the type of epistasis from magnitude to sign for the F145R/M85K pair (compare Figure 6, D/G to Figure 6, H/I). The switch is solely due to the change in the relative energies of the *ca* and *apo* conformations in the *ab* genotype: the *ca* conformation is slightly stabilized relative to the *apo* conformation. The introduction of F145R stabilizes the *apo* conformation, resulting in net stabilization of $\langle G_{ca,\mathrm{apo}}^{Ab}\rangle$. M85K destabilizes both conformations, destabilizing $\langle G_{ca,\mathrm{apo}}^{aB}\rangle$. When both mutations are combined, $\langle G_{ca,\mathrm{apo}}^{AB}\rangle$ is further destabilized, resulting in the observation of sign epistasis (compare pink arrows in Figure 6, H and I).

### Ensemble epistasis is robust to addition of epistasis from structural contacts

We next wanted to ask how the relative magnitude of epistasis changes when we allow epistasis to arise from both the ensemble and structural contacts. We used ROSETTA to calculate the within-conformation interaction energies of 344 mutant pairs. We then re-calculated the stability of each conformation *c* as:

(22) $$G_{c}^{AB}=G_{c}^{ab}+\delta G_{c}^{a\to A}+\delta G_{c}^{b\to B}+\delta \delta G_{c}^{ab\to AB},$$

where $\delta \delta G_{c}^{ab\to AB}$ is the interaction energy within the conformation calculated by ROSETTA. The values of $\delta \delta G_{c}^{ab\to AB}$ had a mean and standard deviation of $9.3\pm 9.8\;{\text{kcal}\cdot \text{mol}}^{-1}$. We used these new values to calculate *ε* in $\langle G_{ca,\mathrm{apo}}\rangle$. Figure 6J shows how the distribution of epistatic magnitude changes when we allow non-additivity to arise from the ensemble alone *vs* both the ensemble and structural contacts. We found that 24% of the 344 mutation pairs exhibit epistasis on the order of $0.6\;{\text{kcal}\cdot \text{mol}}^{-1}$, with an average magnitude of $0.97\;{\text{kcal}\cdot \text{mol}}^{-1}$ when we allow epistasis to arise only from the ensemble. When we allowed epistasis to arise from structural contacts in addition to the ensemble, we found that 35% of pairs exhibited epistasis on the order of $0.6\;{\text{kcal}\cdot \text{mol}}^{-1}$, with an average magnitude of $1.4\;{\text{kcal}\cdot \text{mol}}^{-1}$. The addition of within-conformation contacts widens the distribution relative to the ensemble-only dataset, yielding a modest increase in the average epistatic magnitude. Ensemble epistasis thus seems to be an important source of epistasis, even for proteins that also exhibit epistasis from structural contacts within each conformation.

## Discussion

We found that epistasis can arise from a fundamental property of proteins and other macromolecules: the thermodynamic ensemble. Previously we observed ensemble epistasis using lattice models, but the conditions under which it arises and if they are plausibly met in more realistic models of proteins remained unresolved (Sailer and Harms 2017c). Here we used a simple—but general—thermodynamic model to study the how the ensemble leads to epistasis. Ensemble epistasis arises because mutations can affect any conformation in the ensemble. Since observables are averaged over the entire ensemble, they cannot be separated into additive components.

### Ensemble epistasis should be pervasive in biology

We expect ensemble epistasis in systems where (1) at least three conformations are populated and (2) mutations have differential effects on at least two conformations. The first requirement may be common: multi-conformation ensembles often underlie biological function, from allostery to fold-switching (Figure 2A; Wei *et al.* 2016). The commonality of the second requirement, however, is not as obvious. We tested for the plausibility of meeting the second requirement by modeling the effects of mutations on different conformations of the S100A4 protein. S100A4 is a $Ca^{2+}$ signaling protein that adopts three conformations, meeting the requirement for multiple populated conformations (Figure 5A). We identified mutations that had differential effects on both inactive conformations, which satisfied the second requirement. Nearly half of the mutant pairs exhibited epistasis above $0.6\;{\text{kcal}\cdot \text{mol}}^{-1}$, suggesting that—at least in principle—ensemble epistasis should be detectable in real proteins (Figure 6A).

There is mounting indirect evidence of links between epistasis and thermodynamic ensembles. For example, in TEM-1 *β*-lactamase, two adaptive mutations were identified that independently increased structural heterogeneity and function. Together the mutations exhibited epistasis, shifting the ensemble into a dominantly nonproductive structure (Dellus-Gur *et al.* 2015). Epistasis also underlies changes in dynamics that caused functional divergence between Src and Abl kinases and the evolution of fold-switching proteins (Seeliger *et al.* 2007; Wilson *et al.* 2015).

Recently, a thermodynamic model was used to decompose mutational effects on the GB1 protein (Otwinowski 2018). A three-structure ensemble model was able to explain much of the epistasis observed in the dataset. The remaining epistasis pointed towards residues that contribute to functionally important structural dynamics. This approach yielded mechanistic information about the system. Notably, the mathematical framework of the thermodynamic ensemble is not limited to proteins and other macromolecules—it has been used to describe much more complex biological systems like signaling networks and bacterial communities (Tran *et al.* 2008; Venturi *et al.* 2010; Khazaei *et al.* 2012; Lu *et al.* 2013; Bessonnard *et al.* 2014; Hameri *et al.* 2019).

### Relationship to threshold epistasis

Ensemble epistasis is related to—but conceptually distinct from—threshold epistasis. Threshold epistasis describes non-additivity arising from the accumulation of destabilizing mutations. Below some threshold stability, the fraction of folded protein molecules drops and any function encoded by the folded structure is lost (Bershtein *et al.*, 2006; Bloom *et al.* 2007; Gong *et al.* 2013; Kumar *et al.* 2017; Petrović *et al.* 2018). The same mutation could have no effect on a high stability protein, but be highly deleterious to a low stability protein. Both ensemble and threshold epistasis arise because the protein can populate more than one conformation; however, at this point, the two mechanisms for epistasis diverge.

To make this concrete, consider the activity of an enzyme. Enzyme activity is proportional to the fraction of enzyme molecules that are in the active form. Mutations that have an additive, linear effect on thermodynamic stability will have a nonadditive, nonlinear effect on the fractional population of the active form (Equation 6). As such, we can observe epistasis between mutations at the level of enzyme activity simply because we are describing a nonlinear function (activity) with a linear model (Equation 16; Sailer and Harms 2017a; Otwinowski *et al.* 2018). If we transform the nonlinear fractional population scale (Equation 6) onto a linear free energy scale (Equation 8), threshold epistasis disappears. One can describe the nonadditive, nonlinear effects of mutations on activity as additive, linear effects on stability. This is not to say threshold epistasis does not matter—phenotype and fitness often depend on nonlinear fractional populations—but rather that it is possible to analyze the data in a way that removes epistasis.

Ensemble epistasis, however, cannot be removed by transforming the data onto a linear scale. We describe the observable ($\Delta G_{\mathrm{obs}}$) and the effects of mutations ($\delta G_{c}^{x\to X}$) on the same linear free energy scale. But because mutations have different effects on different conformations, these linear perturbations are re-weighted in nonlinear fashion, thus leading to irreducible epistasis.

### Ensemble epistasis may shape evolution

Though it remains to be seen, we expect that ensemble epistasis plays an important role in shaping protein evolution. We have shown that simple ensembles give rise to magnitude, sign, and reciprocal sign epistasis (Figure 4), and that they may give rise to high-order epistasis (Supplementary Section S3). Sign and reciprocal sign epistasis are particularly important; they can decrease accessible evolutionary trajectories and are required for the presence of multiple peaks in fitness landscapes (Lunzer *et al.* 2005; Weinreich *et al.* 2005, 2006; Bridgham *et al.* 2006; Poelwijk *et al.* 2007, 2011; Kvitek and Sherlock 2011; Salverda *et al.* 2011; Chiotti *et al.* 2014; Palmer *et al.* 2015). High-order epistasis can alter accessibility and can facilitate the bypassing of evolutionary dead-ends in genotype–phenotype maps, making evolution deeply unpredictable (Weinreich *et al.* 2013; Palmer *et al.* 2015; Wu *et al.* 2016; Sailer and Harms 2017b).

Aside from giving rise to evolutionarily relevant classes of epistasis, we anticipate that ensemble epistasis occurs under physiologically relevant—and thus evolutionarily important—conditions. Ensemble epistasis is maximized when multiple conformations are populated (Figure 6A): exactly within the concentration regime where macromolecules act as molecular switches. Further, we found in our S100A4 calculations that we could see changes in the type of epistasis observed as we changed the amount of allosteric effector, $\mu_{Ca^{2+}}$ (Figure 6A). This suggests that ensemble epistasis could play a critical role in shaping the availability of evolutionary trajectories—possibly even in an environment-dependent manner. A small change in the concentration of an effector could open or close new evolutionary trajectories. A similar phenomenon has been observed in allosteric proteins where ligands can act as agonists or antagonists in response to changes in environment, ultimately via changes in the thermodynamic ensemble (Motlagh and Hilser 2012).

### Detecting ensemble epistasis

Our work predicts ensemble epistasis is common. How would one detect it experimentally? Effector- or environment-dependent epistasis may be a signal of ensemble epistasis. One straightforward experimental test for ensemble epistasis would be to perturb the thermodynamic ensemble by tuning environmental factors such as effector concentration (Figure 5B). For S100A4, we observed distinct effector-dependent patterns of epistasis for mutation pairs, where the amount of epistasis we observed changed with the addition of $Ca^{2+}$ (Figure 6A). Ensemble epistasis should be maximized at concentrations where many distinct conformations are populated (*i.e*., at concentrations where functional transitions occur) and minimized when mutations can impact only a single conformation. (*i.e.*, low $\mu_{Ca^{2+}}$). Environmental-dependent epistasis has been noted previously, possibly pointing to an underlying ensemble epistasis (Remold and Lenski 2004; Flynn *et al.* 2013; Chiotti *et al.* 2014; Joshi and Prasad 2014; Barker *et al.* 2015; Samir *et al.* 2015; Guerrero *et al.* 2019; Nosil *et al.* 2020).

Additionally, one might test for ensemble epistasis by measuring the temperature dependence of epistasis. If the free energy of each conformation does not change with temperature, the predictions are straightforward. For very low temperatures, only the deepest energy well—corresponding to the most stable conformation—should be populated, preventing ensemble epistasis. At very high temperature, all conformations will have the same statistical weight, and thus will be equally populated regardless of free energy (Equation 6). But, because of this fact, mutations will not redistribute the populations of the conformations—meaning there will be no ensemble epistasis. For intermediate temperature values, we might expect appreciable temperature-dependent effects on ensemble epistasis. Unfortunately, the free energy of each conformation is not constant with temperature for most proteins (Dill 1990). As such, we would expect the effects of ensemble epistasis are convolved with changes in the enthalpy and entropy of each conformation—making temperature-dependent experiments difficult to interpret.

## Conclusion

Our results reveal that a universal property of proteins and other macromolecules, the thermodynamic ensemble, can lead to epistasis. Although the pervasiveness of ensemble epistasis in biology remains unknown, we anticipate that it is widespread. First, ensemble epistasis is maximized under the physiological conditions where biologically important, ensemble-mediated functions occur. Second, even a simple, three-conformation system can lead to a rich variety of epistasis, suggesting that the necessary conditions for ensemble epistasis are met for many proteins. And, third, structure-based calculations using experimentally solved protein structures revealed the potential for rampant ensemble epistasis. As such, we anticipate that ensemble epistasis plays important roles in shaping protein biology and evolution.

## References

[iyab105-B1] Alexander PA , He Y, Chen Y, Orban J, Bryan PN. 2009. A minimal sequence code for switching protein structure and function. Proc Natl Acad Sci U S A. 106:21149–21154. 19923431 10.1073/pnas.0906408106 PMC2779201

[iyab105-B2] Alford RF , Leaver-Fay A, Jeliazkov JR, O’Meara MJ, DiMaio FP, et al 2017. The Rosetta all-atom energy function for macromolecular modeling and design. J Chem Theory Comput. 13:3031–3048. 28430426 10.1021/acs.jctc.7b00125 PMC5717763

[iyab105-B3] Ancel LW , Fontana W. 2000. Plasticity, evolvability, and modularity in RNA. J Exp Zool. 288:242–283. 11069142 10.1002/1097-010x(20001015)288:3<242::aid-jez5>3.0.co;2-o

[iyab105-B4] Baier F , Hong N, Yang G, Pabis A, Miton CM, et al 2019. Cryptic genetic variation shapes the adaptive evolutionary potential of enzymes. Elife. 8:e40789. 30719972 10.7554/eLife.40789 PMC6372284

[iyab105-B5] Barker B , Xu L, Gu Z. 2015. Dynamic epistasis under varying environmental perturbations. PLoS One. 10:e0114911. 25625594 10.1371/journal.pone.0114911 PMC4308068

[iyab105-B6] Bershtein S , Segal M, Bekerman R, Tokuriki N, Tawfik DS. 2006. Robustness–epistasis link shapes the fitness landscape of a randomly drifting protein. Nature. 444:929–932. 17122770 10.1038/nature05385

[iyab105-B7] Bessonnard S , De Mot L, Gonze D, Barriol M, Dennis C, et al 2014. Gata6, Nanog and Erk signaling control cell fate in the inner cell mass through a tristable regulatory network. Development. 141:3637–3648. 25209243 10.1242/dev.109678

[iyab105-B8] Bloom JD , Arnold FH, Wilke CO. 2007. Breaking proteins with mutations: threads and thresholds in evolution. Mol Syst Biol. 3:76. 17262035 10.1038/msb4100119 PMC1800353

[iyab105-B9] Bridgham JT , Carroll SM, Thornton JW. 2006. Evolution of hormone-receptor complexity by molecular exploitation. Science. 312:97–101. 37000978 10.1681/01.asn.0000926836.46869.e5

[iyab105-B10] Chiotti KE , Kvitek DJ, Schmidt KH, Koniges G, Schwartz K, et al 2014. The Valley-of-Death: reciprocal sign epistasis constrains adaptive trajectories in a constant, nutrient limiting environment. Genomics. 104:431–437. 25449178 10.1016/j.ygeno.2014.10.011 PMC4454348

[iyab105-B11] Dellus-Gur E , Elias M, Caselli E, Prati F, Salverda MLM, et al 2015. Negative epistasis and evolvability in TEM-1 *β*-Lactamase—the thin line between an enzyme’s conformational freedom and disorder. J Mol Biol. 427:2396–2409. 26004540 10.1016/j.jmb.2015.05.011 PMC4718737

[iyab105-B12] Dill KA. 1990. Dominant forces in protein folding. Biochemistry. 29:7133–7155. 2207096 10.1021/bi00483a001

[iyab105-B13] Ecsédi P , Kiss B, Gógl G, Radnai L, Buday L, et al 2017. Regulation of the equilibrium between closed and open conformations of annexin A2 by N-terminal phosphorylation and S100A4-binding. Structure. 25:1195–1207.e5. 28669632 10.1016/j.str.2017.06.001

[iyab105-B14] Flynn KM , Cooper TF, Moore FB-G, Cooper VS. 2013. The environment affects epistatic interactions to alter the topology of an empirical fitness landscape. PLoS Genet. 9:e1003426. 23593024 10.1371/journal.pgen.1003426 PMC3616912

[iyab105-B15] Garrett SC , Hodgson L, Rybin A, Toutchkine A, Hahn KM, et al 2008. A biosensor of S100A4 metastasis factor activation: inhibitor screening and cellular activation dynamics. Biochemistry. 47:986–996. 18154362 10.1021/bi7021624 PMC3227476

[iyab105-B16] Giger L , Caner S, Obexer R, Kast P, Baker D, et al 2013. Evolution of a designed retro-aldolase leads to complete active site remodeling. Nat Chem Biol. 9:494–498. 23748672 10.1038/nchembio.1276 PMC3720730

[iyab105-B17] Gong LI , Suchard MA, Bloom JD. 2013. Stability-mediated epistasis constrains the evolution of an influenza protein. Elife. 2:e00631. 23682315 10.7554/eLife.00631 PMC3654441

[iyab105-B18] Guerrero RF , Scarpino SV, Rodrigues JV, Hartl DL, Ogbunugafor CB. 2019. Proteostasis environment shapes higher-order epistasis operating on antibiotic resistance. Genetics. 212:565–575. 31015194 10.1534/genetics.119.302138 PMC6553834

[iyab105-B19] Hameri T , Boldi M-O, Hatzimanikatis V. 2019. Statistical inference in ensemble modeling of cellular metabolism. PLoS Comput Biol. 15:e1007536. 31815929 10.1371/journal.pcbi.1007536 PMC6922442

[iyab105-B20] Joshi CJ , Prasad A. 2014. Epistatic interactions among metabolic genes depend upon environmental conditions. Mol Biosyst. 10:2578–2589. 25018101 10.1039/c4mb00181h

[iyab105-B21] Khazaei T , McGuigan AP, Mahadevan R. 2012. Ensemble modeling of cancer metabolism. Front Physiol. 135.3:22623918 10.3389/fphys.2012.00135 PMC3353412

[iyab105-B22] Kumar A , Natarajan C, Moriyama H, Witt CC, Weber RE, et al 2017. Stability-mediated epistasis restricts accessible mutational pathways in the functional evolution of avian hemoglobin. Mol Biol Evol. 34:1240–1251. 28201714 10.1093/molbev/msx085 PMC5400398

[iyab105-B23] Kvitek DJ , Sherlock G. 2011. Reciprocal sign epistasis between frequently experimentally evolved adaptive mutations causes a rugged fitness landscape. PLoS Genet. 7:e1002056. 21552329 10.1371/journal.pgen.1002056 PMC3084205

[iyab105-B24] Lu M , Jolly MK, Gomoto R, Huang B, Onuchic J, et al 2013. Tristability in cancer-associated MicroRNA-TF chimera toggle switch. J Phys Chem B. 117:13164–13174. 23679052 10.1021/jp403156m

[iyab105-B25] Lunzer M , Miller SP, Felsheim R, Dean AM. 2005. The biochemical architecture of an ancient adaptive landscape. Science. 310:499–501. 16239478 10.1126/science.1115649

[iyab105-B26] Maisnier-Patin S , Paulander W, Pennhag A, Andersson DI. 2007. Compensatory evolution reveals functional interactions between ribosomal proteins S12, L14 and L19. J Mol Biol. 366:207–215. 17157877 10.1016/j.jmb.2006.11.047

[iyab105-B27] Malashkevich VN , Varney KM, Garrett SC, Wilder PT, Knight D, et al 2008. Structure of Ca2+-bound S100A4 and its interaction with peptides derived from nonmuscle myosin-IIA. Biochemistry. 47:5111–5126. 18410126 10.1021/bi702537s PMC2633413

[iyab105-B28] Miton CM , Tokuriki N. 2016. How mutational epistasis impairs predictability in protein evolution and design. Protein Sci. 25:1260–1272. 26757214 10.1002/pro.2876 PMC4918425

[iyab105-B29] Motlagh HN , Hilser VJ. 2012. Agonism/antagonism switching in allosteric ensembles. Proc Natl Acad Sci U S A. 109:4134–4139. 22388747 10.1073/pnas.1120519109 PMC3306695

[iyab105-B30] Motlagh HN , Wrabl JO, Li J, Hilser VJ. 2014. The ensemble nature of allostery. Nature. 508:331–339. 24740064 10.1038/nature13001 PMC4224315

[iyab105-B31] Nosil P , Villoutreix R, de Carvalho CF, Feder JL, Parchman TL, et al 2020. Ecology shapes epistasis in a genotype–phenotype–fitness map for stick insect colour. Nat Ecol Evol. 1673–1684. 4:32929238 10.1038/s41559-020-01305-y

[iyab105-B32] Ortlund EA , Bridgham JT, Redinbo MR, Thornton JW. 2007. Crystal structure of an ancient protein: evolution by conformational epistasis. Science. 317:1544–1548. 17702911 10.1126/science.1142819 PMC2519897

[iyab105-B33] Otwinowski J. 2018. Biophysical inference of epistasis and the effects of mutations on protein stability and function. Mol Biol Evol. 35:2345–2354. 30085303 10.1093/molbev/msy141 PMC6188545

[iyab105-B34] Otwinowski J , McCandlish DM, Plotkin JB. 2018. Inferring the shape of global epistasis. Proc Natl Acad Sci U S A. 115:E7550–E7558. 30037990 10.1073/pnas.1804015115 PMC6094095

[iyab105-B35] Palmer AC , Toprak E, Baym M, Kim S, Veres A, et al 2015. Delayed commitment to evolutionary fate in antibiotic resistance fitness landscapes. Nat Commun. 6:7385. 26060115 10.1038/ncomms8385 PMC4548896

[iyab105-B36] Park H , Bradley P, Greisen P, Liu Y, Mulligan VK, et al 2016. Simultaneous optimization of biomolecular energy functions on features from small molecules and macromolecules. J Chem Theory Comput. 12:6201–6212. 27766851 10.1021/acs.jctc.6b00819 PMC5515585

[iyab105-B37] Petrović D , Risso VA, Kamerlin SCL, Sanchez-Ruiz JM. 2018. Conformational dynamics and enzyme evolution. J R Soc Interface. 15:20180330. 30021929 10.1098/rsif.2018.0330 PMC6073641

[iyab105-B38] Poelwijk FJ , Kiviet DJ, Weinreich DM, Tans SJ. 2007. Empirical fitness landscapes reveal accessible evolutionary paths. Nature. 445:383–386. 17251971 10.1038/nature05451

[iyab105-B39] Poelwijk FJ , Tănase-Nicola S, Kiviet DJ, Tans SJ. 2011. Reciprocal sign epistasis is a necessary condition for multi-peaked fitness landscapes. J Theor Biol. 272:141–144. 21167837 10.1016/j.jtbi.2010.12.015

[iyab105-B40] Remold SK , Lenski RE. 2004. Pervasive joint influence of epistasis and plasticity on mutational effects in *Escherichia coli*. Nat Genet. 36:423–426. 15072075 10.1038/ng1324

[iyab105-B41] Sailer ZR , Harms MJ. 2017a. Detecting high-order epistasis in nonlinear genotype-phenotype maps. Genetics. 205:1079–1088. 28100592 10.1534/genetics.116.195214 PMC5340324

[iyab105-B42] Sailer ZR , Harms MJ. 2017b. High-order epistasis shapes evolutionary trajectories. PLoS Comput Biol. 13:e1005541. 28505183 10.1371/journal.pcbi.1005541 PMC5448810

[iyab105-B43] Sailer ZR , Harms MJ. 2017c. Molecular ensembles make evolution unpredictable. Proc Natl Acad Sci U S A. 114:11938–11943. 29078365 10.1073/pnas.1711927114 PMC5691298

[iyab105-B44] Salverda MLM , Dellus E, Gorter FA, Debets AJM, van der Oost J, et al 2011. Initial mutations direct alternative pathways of protein evolution. PLoS Genet. 7:e1001321. 21408208 10.1371/journal.pgen.1001321 PMC3048372

[iyab105-B45] Samir P , Rahul, Slaughter JC, Link AJ. 2015. Environmental interactions and epistasis are revealed in the proteomic responses to complex stimuli. PLoS One. 10:e0134099. 26247773 10.1371/journal.pone.0134099 PMC4527715

[iyab105-B46] Seeliger MA , B, Nagar F, Frank X, Cao MN, Henderson, et al 2007. c-Src binds to the cancer drug imatinib with an inactive Abl/c-Kit conformation and a distributed thermodynamic penalty. Structure. 15:299–311. 17355866 10.1016/j.str.2007.01.015

[iyab105-B47] Sykora J , Brezovsky J, Koudelakova T, Lahoda M, Fortova A, et al 2014. Dynamics and hydration explain failed functional transformation in dehalogenase design. Nat Chem Biol. 10:428–430. 24727901 10.1038/nchembio.1502

[iyab105-B48] Tran LM , Rizk ML, Liao ML 2008. Ensemble modeling of metabolic networks. Biophys J. 95:5606–5617. 18820235 10.1529/biophysj.108.135442 PMC2599852

[iyab105-B49] Tsai C-J , Nussinov R. 2014. A unified view of “How Allostery Works”. PLOS Comput Biol. 10:e1003394. 24516370 10.1371/journal.pcbi.1003394 PMC3916236

[iyab105-B50] Vallely KM , Rustandi RR, Ellis KC, Varlamova O, Bresnick AR, et al 2002. Solution structure of human Mts1 (S100A4) as determined by NMR spectroscopy. Biochemistry. 41:12670–12680. 12379109 10.1021/bi020365r

[iyab105-B51] Venturi V , Kerényi Á, Reiz B, Bihary D, Pongor S. 2010. Locality versus globality in bacterial signalling: can local communication stabilize bacterial communities? Biol Direct. 5:30. 20423483 10.1186/1745-6150-5-30 PMC2873267

[iyab105-B52] Wei G , Xi W, Nussinov R, Ma B. 2016. Protein ensembles: how does nature harness thermodynamic fluctuations for life? The diverse functional roles of conformational ensembles in the cell. Chem Rev. 116:6516–6551. 26807783 10.1021/acs.chemrev.5b00562 PMC6407618

[iyab105-B53] Weinreich DM , Delaney NF, Depristo MA, Hartl DL. 2006. Darwinian evolution can follow only very few mutational paths to fitter proteins. Science. 312:111–114. 16601193 10.1126/science.1123539

[iyab105-B54] Weinreich DM , Lan Y, Wylie CS, Heckendorn RB. 2013. Should evolutionary geneticists worry about higher-order epistasis? Curr Opin Genet Dev. 23:700–707. 24290990 10.1016/j.gde.2013.10.007 PMC4313208

[iyab105-B55] Weinreich DM , Watson RA, Chao L. 2005. Perspective: sign epistasis and genetic costraint on evolutionary trajectories. Evolution. 59:1165–1174. 16050094

[iyab105-B56] Wilson C , Agafonov RV, Hoemberger M, Kutter S, Zorba A, et al 2015. Using ancient protein kinases to unravel a modern cancer drug’s mechanism. Science. 347:882–886. 25700521 10.1126/science.aaa1823 PMC4405104

[iyab105-B57] Wu NC , Dai L, Olson CA, Lloyd-Smith JO, Sun R. 2016. Adaptation in protein fitness landscapes is facilitated by indirect paths. Elife. 5:e16965. 27391790 10.7554/eLife.16965 PMC4985287

[iyab105-B58] Yang G , Anderson DW, Baier F, Dohmen E, Hong N, et al 2019. Higher-order epistasis shapes the fitness landscape of a xenobiotic-degrading enzyme. Nat Chem Biol. 15:1120–1128. 31636435 10.1038/s41589-019-0386-3

[iyab105-B59] Yokoyama S , Xing J, Liu Y, Faggionato D, Altun A, et al 2014. Epistatic adaptive evolution of human color vision. PLOS Genet. 10:e1004884. 25522367 10.1371/journal.pgen.1004884 PMC4270479

